# Molecular and restriction fragment length polymorphism analysis of canine parvovirus 2 (CPV-2) in dogs in southeast Anatolia, Turkey

**DOI:** 10.4102/ojvr.v86i1.1734

**Published:** 2019-08-27

**Authors:** Pelin F. Polat, Adem Şahan, Gürbüz Aksoy, Mehmet O. Timurkan, Ender Dinçer

**Affiliations:** 1 Department of Internal Medicine, Faculty of Veterinary Medicine, Harran University, Sanliurfa, Turkey; 2 Department of Virology, Faculty of Veterinary Medicine, Atatürk University, Erzurum, Turkey; 3 Advanced Technology Education, Research and Application Center, Mersin University, Mersin, Turkey

**Keywords:** Canine Parvovirus variants, molecular characterisation, Turkey, RFLP, restriction fragment length polymorphism

## Abstract

Canine parvovirus-2 (CPV-2) is the aetiological agent of an infectious viral disease of dogs, characterised by diarrhoea and vomiting. Mutations of the CPV-2 genome have generated new variants circulating worldwide. This article reports the molecular analysis of CPV-2 variants collected in the dog population in southeast Anatolia, Turkey. Twenty blood samples previously taken for the laboratory diagnosis of dogs with suspected parvovirus were screened for CPV-2 by polymerase chain reaction (PCR). Of the 20 samples, 18 tested positive for CPV-2. Partial VP2 gene sequencing and restriction fragment length polymorphism (RFLP) analysis revealed CPV-2a (*n* = 1), CPV-2b (*n* = 16) and CPV-2c (*n* = 1) variants. Phylogenetic analysis based on the partial length VP2 gene showed that CPV-2b (*n* = 15) variants showed sequences clustering separately in the phylogenetic tree. The CPV-2c sample was phylogenetically related to Chinese strains and Indonesia strain, whereas the CPV-2a sample was phylogenetically related to the Portuguese strain. These results, which are the first to demonstrate the presence of CPV-2c in the dog population of southeast Anatolia, Turkey, indicate that CPV-2a/2b/2c variants co-exist in Turkey’s dog population.

## Introduction

Canine parvovirus type 2 (CPV-2) causes an infectious viral disease of dogs, characterised by diarrhoea, vomiting and heart failure in pups. Although its origin is still unknown, CPV-2 is probably derived from feline panleukopenia virus (FPV) or FPV-like carnivore parvovirus, which is widespread worldwide with different frequencies (Calderon et al. 2009; Hayes et al. 1979; Mittal et al. 2014; Nandi & Kumar 2010) CPV-2 (now included in the species *Carnivore protoparvovirus 1*) is a non-enveloped, single-stranded Deoxyribonucleic acid (DNA) virus of genus *Protoparvovirus*, subfamily *Parvovirinae*, and family *Parvoviridae* (Catmore et al. 2019). The virus has a 5.2 kb genome with two major open reading frames (ORFs). One ORF encodes for the VP1 and VP2 capsid proteins, while the other ORF expresses non-structural proteins (NS1 and NS2). The VP2 capsid protein is the most important viral determinant for host range. Amino acid mutations of this protein have important biological consequences, such as canine–feline host range and antigenic properties (Calderon et al. 2009; Dei Giudici et al. 2017; Mira et al. 2017; Muz et al. 2012).

In the late 1970s, CPV-2 emerged to become widespread in dog populations worldwide. After an adaptation period, CPV-2 caused a pandemic in dogs in 1978–1980. New antigenic variants, named CPV-2a and CPV-2b, resulted from mutations in CPV-2. In 2001, a new antigenic variant, CPV-2c, with amino acid substitution 426 (Asp→Glu) in the VP2 capsid protein, was found in Italy (Battilani et al. 2001; Decaro & Buonavoglia 2012). These three variants can be distinguished by only one amino acid change at residue 426 (Asn in CPV-2a, Asp in CPV-2b and Glu in CPV-2c) (Pérez et al. 2012), using polymerase chain reaction (PCR)-based genotyping assay and sequencing (Chou et al. 2011). These three variants are separated from the original CPV-2 by five to six amino acid residues (Battilani et al. 2001). During the 2000s, a change to residue 297 (Ser→Ala) created two new variants, reported as a new CPV-2a/2b (Battilani et al. 2001). The distribution of CPV-2 variants varies between countries, and the CPV-2a/2b/2c variants currently circulate at different rates. For example, recent epidemiological investigations have revealed that CPV-2a is the main variant circulating in Australia (Woolford et al. 2017), India (Nandi & Kumar 2010), Korea (Geng et al. 2015) and Greece (Ntafis et al. 2010), while CPV-2b has been reported in Taiwan (Chou et al. 2011), Italy (Dei Giudici et al. 2017; Mira et al. 2018; Tucciarone et al. 2018), South Africa, North America, Greece (Ntafis et al. 2010) and Iraq (Sheikh et al. 2017). The CPV-2c variant has also been identified in Spain, the United States (US), Portugal, Germany (Decaro &Buonavoglia 2012; Pérez et al. 2012), Argentina (Calderon et al. 2009), Uruguay (Pérez et al. 2012) and Sweden (Sutton et al. 2013). Finally, all three variants have been found co-circulating in Tunisia and Greece (Decaro & Buonavoglia 2012).

Vaccination is the only protection from the disease, and inactivated and modified live virus vaccines have recently been used to immunise dogs (Zhou et al. 2017). New mutations (Y324I and T440A) have recently emerged on the VP2 protein because of antigenic drift in the global dog population. The presence of antigenic variants has raised concerns about the efficacy of existing vaccines because of the risk of vaccine failure (Pratelli et al. 2001; Zhou et al. 2017). In Turkey, CPV vaccines (Nobivac – Intervet; Vanguard – Pfizer; Parvodog – Merial; and Quantum – Schering) with 2a and 2b variants have been used to immunise the dog population.

**FIGURE 1:**
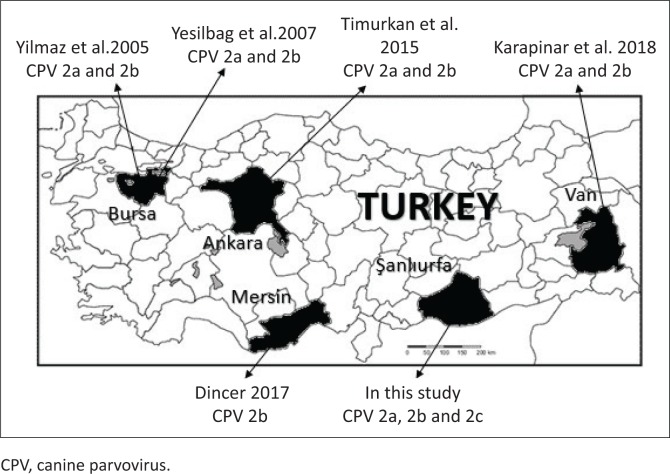
Illustrative map of canine parvovirus-2 variant (2a/2b/2c) locations in the studies.

There have been few molecular characterisation studies of CPV-2 variants in Turkey (Table 1 and Figure 1). Some studies have reported that CPV-2a/2b is present in dogs (Karapınar, Dincer & Ozkan 2018; Timurkan & Oguzoğlu 2015; Yesilbag et al. 2007; Yılmaz, Pratelli & Torun 2005), while CPV-2c has been detected in the blood samples of cat collected from Ankara Province (Muz et al. 2012). However, there are no reports yet of CPV-2c in Turkey’s dog population.

**TABLE 1:** Studies of canine parvovirus -2 genotyping of dogs in Turkey.

Study year	Regions of Turkey (province)	Variants of CPV - 2			Methods	References
		2a	2b	2c		
2003	Bursa	9	7	-	HI	Yılmaz et al. 2005
2002–2003	Bursa	17	10	-	HI	Yesilbag et al. 2007
2009–2010	Ankara	17	8	-	PCR, Sequence	Timurkan and Oguzoğlu 2015
2017	Mersin	-	8	-	PCR, Sequence	Dincer 2017
2018	Van	1	3	-	PCR, Sequence	Karapınar, Dincer and Ozkan 2018
2017	Sanliurfa	1	16	1	PCR–RFLP, Sequence	In this study

HI, haemagglutination inhibition; PCR, polymerase chain reaction; PCR–RFLP, polymerase chain reaction–restriction fragment length polymorphism.

The aim of this study was to characterise CPV-2 variants, using PCR and restriction fragment length polymorphism (RFLP) methods, from clinically ill dog samples in southeast Anatolia, Turkey (Sanliurfa Province).

## Material and methods

### Samples

Blood samples (*n* = 20) from dogs with gastroenteritis were investigated. These dogs were submitted to the Department of Internal Medicine (Faculty of Veterinary Medicine, Harran University, Turkey) by citizens from the centre and other districts of Sanliurfa Province in 2017. Only blood (taken for blood gas analysis and leucocyte level) samples were taken for diagnosis of the disease. No stool samples were taken. The clinical samples were stored at –20 °C until used in the study. Because these blood samples were collection material, no ethical approval was required. Clinical symptoms in the sampled dogs included fever, anorexia, lethargy, vomiting, severe bloody diarrhoea, severe weight loss and leucopenia. All the sampled dogs were strays without any vaccination history. Information about each dog is given in Table 2.

**TABLE 2:** Sample no, age, accession numbers and clinical sings of dogs infected with canine parvovirus.

Sample no.	Sample type	Age (month)	Sex	Vaccination status	Clinical remark	Breed	Year	Type	Accession number
1	Blood	2.5	F	NV	Anorexia and diarrhoea	Mix	2017	2b	MG780275
2	Blood	3	M	NV	Diarrhoea	Mix	2017	2b	MG780276
3	Blood	2	F	NV	Diarrhoea	Mix	2017	2b	MG780277
5	Blood	5	M	NV	Anorexia and depression	Mix	2017	2b	MG780278
11	Blood	2	M	NV	Vomiting	Mix	2017	2b	MG780279
12	Blood	1.5	M	NV	Vomiting and diarrhoea	Mix	2017	2b	MG780280
13	Blood	2	M	NV	Anorexia and vomiting	Mix	2017	2b	MG780281
15	Blood	3	F	NV	Anorexia and vomiting	Mix	2017	2c	MG780282
16	Blood	2.5	M	NV	Diarrhoea	Mix	2017	2b	MG780283
17	Blood	1.5	M	NV	Diarrhoea	Mix	2017	2b	MG780284
18	Blood	3	F	NV	Anorexia and diarrhoea	Mix	2017	2b	MG780285
66	Blood	3	F	NV	Anorexia and depression	Mix	2017	2b	MG780286
70	Blood	2	M	NV	Anorexia, anaemia and depression	Mix	2017	2b	MG780287
100	Blood	3	F	NV	Diarrhoea	Mix	2017	2a	MG780288
101	Blood	2	M	NV	Anorexia and diarrhoea	Mix	2017	2b	MG780289
102	Blood	3	M	NV	Anorexia and diarrhoea	Mix	2017	2b	MG780290
110	Blood	1.5	F	NV	Diarrhoea	Mix	2017	2b	MG780291
189	Blood	4	F	NV	Diarrhoea	Mix	2017	2b	MG780292

F, female; M, male; NV, non-vaccinated.

### Deoxyribonucleic acid extraction, polymerase chain reaction and restriction fragment length polymorphism analysis

CPV-2 genomic DNA was extracted from clinical specimens using a High Pure Viral Nucleic Acid Kit (Roche Diagnostics, Mannheim, Germany) following the manufacturer’s recommendations. Purified viral genomic DNA was eluted in 50 *µ*L of elution buffer and then stored at –20 °C until PCR was performed.

The PCR was performed using Hfor and Hrev primers for detecting the partial VP2 gene (630 base pairs [bp]) of CPV-2, according to the protocol reported elsewhere (Battilani et al. 2001). The amplified PCR products were visualised using 1% agarose gel electrophoresis stained with ethidium bromide. The size of the PCR amplicons was determined using the 100 bp marker (DNA ladder, Thermo Fisher Scientific, US).

**FIGURE 2:**
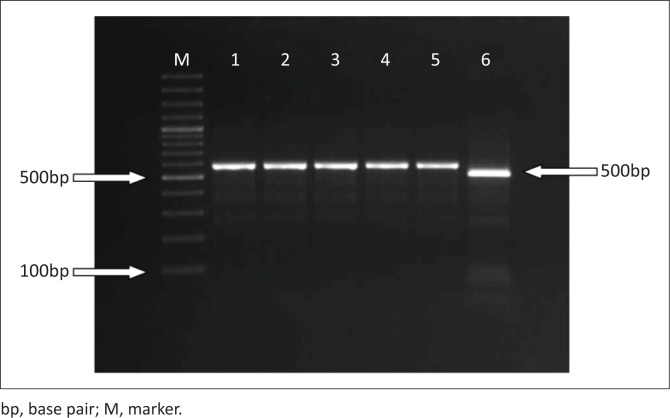
Restriction fragment length polymorphism analyses of the partial VP2 gene polymerase chain reaction amplicon (583 base pairs) of canine parvovirus-2a/2b/2c variants. Line M: 100 base pairs Deoxyribonucleic acid ladder (Thermoscientific, United States); Line 1: undigested canine parvovirus- 2a; Line 2: undigested canine parvovirus-2b; Line 3: undigested canine parvovirus-2c; Line 4; canine parvovirus-2a undigested with *Mbo*II; Line 5: canine parvovirus-2b undigested with *Mbo*II; Line 6: canine parvovirus-2c digested with *Mbo*II.

For RFLP analysis, viral genomic DNA was amplified using 555for-5’-(CAGGAAGATATCCAGAAGGA)-3’ (from 4003 to 4022)/555rev-5’-(GGTGCTAGTTGATATGTAATAAACA)-3’ (from 4585 to 4561) primers to obtain the partial VP2 gene, region (583nt) (Buonavoglia et al. 2001). The amplified PCR amplicons were then digested using the enzyme *Mbo*II (FastDigest, Thermo Fisher Scientific, US) for RFLP characterisation, which distinguished CPV-2a/2b variants from CPV-2c (Buonavoglia et al. 2001). The 583-bp PCR amplicons were digested with 2 *µ*L (5U/1 *µ*L) of the restriction enzyme *Mbo*II, 1 *µ*L of 10x *Mbo*II buffer and 8 *µ*L of molecular grade water for 1 hour at 37 °C on a heat block. Subsequently, digested and non-digested amplicons were assessed on 1.5% agarose gel electrophoresis stained with ethidium bromide (Figure 2).

### Deoxyribonucleic acid sequencing and comparative analysis

Sequence analysis was performed on all positive samples. The PCR amplicons were cleaned with a GeneJet PCR Purification Kit (Thermo Fisher Scientific, US) before sequencing in an ABI PRISM 310 Genetic Analyzer (Applied Biosystem, CA, US) with Hfor and Hrev primers for PCR. The sequence data were submitted to the DDBJ/EMBL/GenBank databases under the following accession numbers: *MG780275–MG780292* (Table 2). The acquired DNA sequences were compared with other CPV reference strains available from GenBank Database (http://www.ncbi.nlm.nih.gov). Phylogenetic analysis included three canine (Nobivac, Intervet; Vanguard, Pfizer; Quantum, Schering) and two feline (Felocell, Pfizer; Purevax, Merial) vaccine strains and one FPV (accession no. M38246). The bioedit program was used for nucleotide and amino acid alignment comparisons (http://www.mbio.ncsu.edu/BioEdit/bioedit.html) (Hall 1999). Phylogenetic analyses were conducted with the neighbour-joining method, using MEGA6 (Tamura et al. 2013).

### Ethical considerations

This article followed all ethical standards for research without direct contact with human or animal subjects.

## Results

**FIGURE 3:**
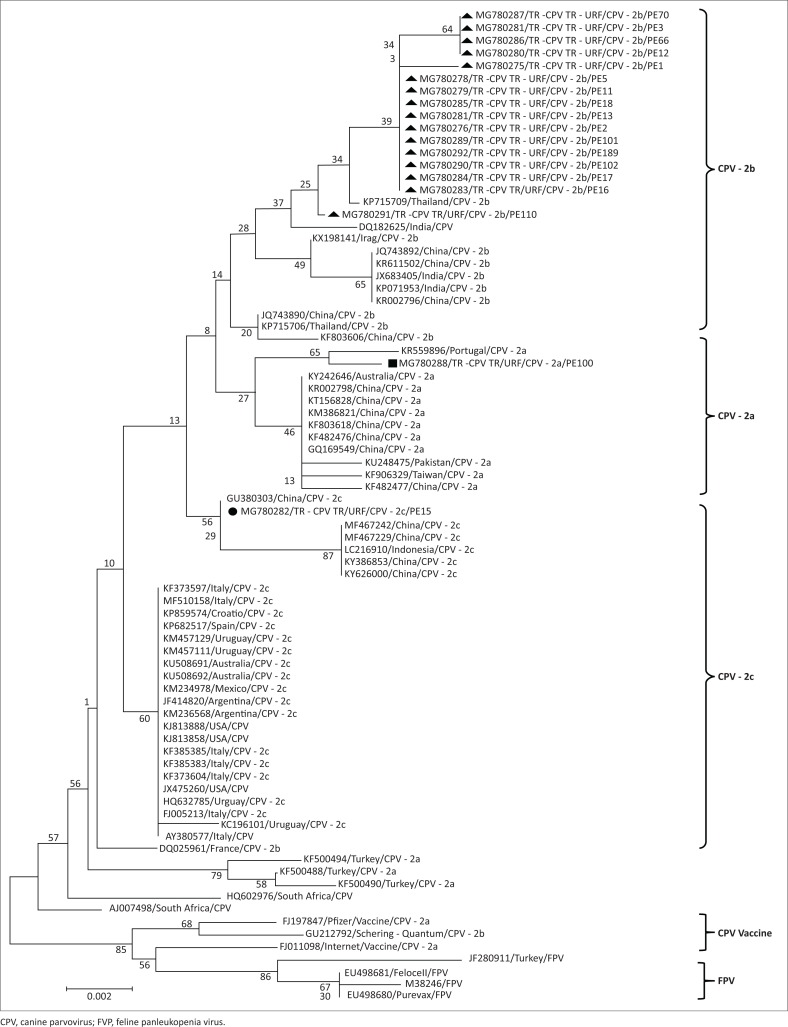
Phylogenetic tree based on the partial VP2 gene of canine parvovirus-2 shows canine parvovirus variants circulating in Sanliurfa, Turkey, and reference sequences. The phylogeny was performed neighbour-joining method based on 1000 replicates by MEGA 6 software. Each sequence is indicated with accession number, country and canine parvovirus-2 variants. In this study, canine parvovirus-2a sequence is indicated with ‘*square*’, canine parvovirus-2b sequence is indicated with *‘triangle*’ and canine parvovirus-2c sequence is indicated with ‘*circle*’.

Twenty blood specimens from dogs showing signs of gastroenteritis (bloody diarrhoea, vomiting, etc.) were tested for CPV-2 using PCR and RFLP. Of these samples, 18 were identified by PCR as positive for CPV-2, whereas 2 samples were negative. Restriction fragment length polymorphism and sequence analysis of the PCR products revealed that only one sample was positive for CPV-2c; the other samples were positive for CPV-2a and CPV-2b (Figures 2 and 3).

The dogs were aged between 1.5 and 5 months; 40% (8/20) were female and 60% (12/20) were male. According to the clinical data, the dogs had not been known previously vaccinated. All dogs were of mixed breed.

The partial VP2 gene segment of CPV-2 was obtained using Hfor and Hrev oligonucleotides from the buffy coat (*n* = 18) of the dog samples. Obtained PCR amplicons were sequenced using the same primer set. The sequence analysis revealed amino acid divergences between the sequences analysed in this study and related samples from GenBank at residues 297, 300, 305, 316, 324, 375 and 440 (Table 3). Sequence analysis results showed that 16 samples were characterised as CPV-2b, 1 was characterised as CPV-2a and 1 was characterised as CPV-2c, according to the 426 residue of the VP2 protein amino acid sequence (Table 3). The nucleotide similarity (homology) of our variants was 99.0% – 100.0% for the Turkish strains and 97.8% – 99.0% for the other world strains. No co-infections by multiple CPV-2 variants were detected in the investigated samples. The phylogenetic tree (Figure 3) revealed that (1) 15 of the TR-URF/CPV-2b variants formed a separate group within the reference CPV-2b sequences; (2) the TR-URF/CPV-2a variant was more similar to the Portuguese strain (accession no. KR559896); and (3) the TR-URF/CPV-2c variant was more similar to Chinese strains (accession no.GU380303, MF467242, MF467229, KY386853 and KY626000) and the Indonesia strain (accession no. LC216910). Finally, comparison of the 18 Turkish CPV sequences to the 3 vaccine strains revealed that the vaccine strains were from a separate branch.

**TABLE 3a:** Amino acid changes in VP2 partial gene of canine parvovirus-2a, canine parvovirus-2b and canine parvovirus-2c.

Aa no.	297	300	305	316	323	324	336	375	426	440
M38245	S	A	D	V	N	Y	V	N	N	T
MG780275	A	G	Y	-	-	I	-	D	D	A
MG780266	A	G	Y	-	-	I	-	D	D	A
MG780277	A	G	Y	-	-	I	-	D	D	A
MG780278	A	G	Y	-	-	I	-	D	D	A
MG780279	A	G	Y	-	-	I	-	D	D	A
MG780280	A	G	Y	-	-	I	-	D	D	A
MG780281	A	G	Y	-	-	I	-	D	D	A
MG780282	A	G	Y	-	-	I	-	D	E	-
MG780282	A	G	Y	-	-	I	-	D	D	A
MG780283	A	G	Y	-	-	I	-	D	D	A
MG780284	A	G	Y	-	-	I	-	D	D	A
MG780285	A	G	Y	-	-	I	-	D	D	A
MG780286	A	G	Y	-	-	I	-	D	D	A
MG780287	A	G	Y	-	-	I	-	D	-	-
MG780288	A	G	Y	-	-	I	-	D	D	A
MG780280	A	G	Y	-	-	I	-	D	D	A
MG780290	A	G	Y	-	-	I	-	D	D	A
MG780291	A	G	Y	-	-	I	-	D	D	A
FJ197847	S	-	-	-	-	-	-	E	-	-
GU212792	S	-	-	-	-	-	-	E	D	-
FJ011098	-	-	-	I	-	-	-	-	-	-

Note: Homologous amino acid by sign (−).

**TABLE 3b:** Amino acid changes in VP2 partial gene of canine parvovirus-2a, canine parvovirus-2b and canine parvovirus-2c.

Aa no.	297	300	305	316	323	324	336	375	426	440
aa change	TCT→GCT	GCT→GGT	GAT→TAT	-	AAT→GAT	TAT→ATT	GTA→GTG	AAT→GAT	ATT→GAT	ACA→GCA
	S→A†	A→G†	D→Y†	-	N→N	Y→I†	V→V	N→D†	ATT→GAA	T→A†
	-	-	-	-	-	-	-	-	AAT→AAT	-
	-	-	-	-	-	-	-	-	N→D†	-
	-	-	-	-	-	-	-	-	N→ E†	-
	-	-	-	-	-	-	-	-	N→ N†	-

Note: Amino acid substitutions are indicated by dagger (†) and vaccine strains (FJ197847, GU212792, FJ011098). Homologous amino acid by sign (−).

In this study, some amino acid changes were observed at residues 297, 300, 305, 324, 375, 426 and 440 compared to the reference genes (accession number: M38245) (Table 3). We detected 297 (Ser→Ala), 300 (Ala→Gly), 305 (Asp→Tyr), 324 (Tyr→Ile) and 375 (Asn→Asp) substitutions in all CPV-2a/2b/2c variants compared to the reference strain. We also found substitutions at 426 (Asn→Asp) as CPV-2a, (Asn→Asn) as CPV-2b and (Asn→Glu) as CPV-2c.

## Discussion

Canine parvovirus type 2, an important viral agent of domestic and wild canids, causes haemorrhagic gastroenteritis and myocardial disease, especially in the young. Although CPV-2 has a DNA genome, it displays high rates of nucleotide changes leading to the emergence of new variants (2a/2b/2c) (Shackelton et al. 2005; Pérez et al. 2012). Canine parvovirus-2a/2b variants are prevalent in both Asian countries, such as China, Korea, India and Japan (Geng et al. 2015; Zhao et al. 2016), and European countries, such as Italy (Dei Giudici et al. 2017), Greece (Ntafis et al. 2010), Portugal (Miranda & Thompson 2016) and Switzerland (Nandi & Kumar 2010). The CPV-2c variant has been reported on several continents, including Europe (Portugal, Greece, ltaly, Spain, France, Belgium, Sweden and the United Kingdom) (Decaro & Buonavoglia 2012; Decora et al. 2006; Mira et al. 2017; Ntafis et al. 2010; Sutton et al. 2013), Africa (Tunisia), South America (Uruguay, Brazil and Argentina) (Calderon et al. 2009), Australia (Woolford et al. 2017) and Asia (Geng et al. 2015; Nandi & Kumar 2010; Sheikh et al. 2017).

This study provides the first molecular and sequence analysis of CPV-2 strains from the dog population in southeast Anatolia, Turkey. According to Demeter et al. (2010), *Mbo*II-based RFLP analysis is misleading for detection of CPV-2c because CPV-2a can show the same RFLP results as CPV 2c. However, Figueiredo et al. (2017) showed that CPV-positive PCR amplicons were not digested with *Mbo*II enzyme and identified as CPV-2/2a/2b. This suggests that *Mbo*II-based RFLP analysis is unreliable if used alone to identify CPV-2 variants in samples. Instead, RFLP analysis can be used in conjunction with sequence analysis for the characterisation of CPV-2 variants. Nucleic acid-based techniques (conventional and real-time PCR) have been developed to differentiate the old variant strains used in vaccines from new variants. In addition, PCR–RFLP assay has recently provided a time-saving and low-cost method for detecting and characterising CPV-2 variants in dog samples (Chou et al. 2011). However, molecular techniques, RFLP and PCR–RFLP may not enable molecular typing of clinical samples (Parthiban et al. 2010), so sequence analysis is required to accurately distinguish the CPV variant from indicator amino acid residues (Castro et al. 2011). Therefore, recent studies have used sequencing and RFLP analysis together to provide greater precision in the molecular typing of variants.

In Turkey, the CPV-2c variant has only been demonstrated in cats in Ankara Province (Muz et al. 2012), while CPV-2 variants in Turkey have only been characterised for a few provinces (Dincer 2017; Karapınar et al. 2018; Timurkan & Oguzoğlu 2015; Yesilbag et al. 2007). One study of Mersin Province found that CPV-2b was the major variant in CPV-infected dogs (Dincer 2017), which is in line with our results (16/18 [88.8%] of our samples were CPV-2b). On the other hand, other studies have detected more CPV-2a than CPV-2b samples in infected dogs. Muz et al. (2012) found that CPV-2c obtained from one cat had the amino acid residues 297S, 300A, 305D, 311N, 323D and 324Y. These changes have also been found in the corresponding reference sequence for FPLV (GeneBank no: M36246). However, we detected amino acid residues 297A, 300G, 305Y, 311D, 323N and 324I, which indicate that these two variants may be quite different from each other. To better understand this, a larger gene region will need to be examined in Turkey to determine the origin viruses precisely.

The VP2 protein, which has amino acid residues at 87, 101, 297, 300, 305, 323, 324, 375 and 440, is a major antigenic determinant that plays a pivotal role in the host distribution of the virus and an important role in modulating host response. Additionally, changes in the VP2 protein amino acid residues may increase pathogenicity (Dei Giudici et al. 2017; Muz et al. 2012). In this study, we detected amino acid residues substitutions on the VP2 protein at 297, 300, 305, 323, 324, 426, 375 and 440 (Table 3). We also showed differences in the amino acid residues between the three canine vaccines and the Sanliurfa Province field sequences, including 297, 300, 305, 316, 324, 375 and 440. In Iraq, CPV-2b isolates obtained from dogs have demonstrated similar differences in the VP2 protein amino acid residues (Sheikh et al. 2017). These changes may both reduce vaccine efficacy and increase the pathogenicity of CPV-2 variants. In particular, residue 324I shows strong positive selection in all carnivore parvoviruses (Lin et al. 2014). Residue 323, which lies adjacent to Y324I, plays a role in host range as previously reported (Dei Giudici et al. 2017; Yılmaz et al. 2005). Although the function of residue Y324I has not been fully determined, Lin et al. (2014) reported that its mutation was responsible of viral shedding for up to 2 months in ill dogs. Recently, amino acid changes have been reported in field isolates of CPV-2 in Turkey, excluding amino acid residue Y324I, in dogs (Dincer 2017; Timurkan & Oguzoğlu 2015) and cats (Muz et al. 2012). In this study, we detected the 324I mutation in all our CPV-2a/2b/2c variants, and to the best of our knowledge, only Dincer (2017) has previously reported such a change in Mersin Province, Turkey. In addition, the 324I mutation, which is common in Asian CPV-2 variants, has been found in the isolates from Taiwan (Lin et al. 2014), India and Japan (Geng et al. 2015). In Europe, the 324I mutation has been reported in CPV-2a isolates from Hungary (Cságola et al. 2014). Residue 440A, which is important antigenically and varies within the CPV-2 variants, is located in the VP2 protein GH loop (Muz et al. 2012). The T440A mutation has been reported in Italian CPV-2a (Dei Giudici et al. 2017), Korean CPV-2a (Yoon et al. 2009), Taiwanese CPV-2a (Chiang et al. 2016), Italian CPV-2b (Dei Giudici et al. 2017) and Argentine and Italian CPV-2c (Dei Giudici et al. 2017; Calderon et al. 2009). Recent studies have reported 440A mutation in the dog population in Turkey (Muz et al. 2012; Timurkan & Oguzoğlu 2015). We also detected 440A mutation in CPV-2a and CPV-2c variants in our dog samples. This indicates that further studies of 440A and 324I are needed to better understand the relationship between this mutation and the severity of clinical symptoms.

Previous studies have reported that CPV-2 variants (CPV-2a/2b/2c) can infect and cause disease in cats through transfer from dogs, although cat-to-cat transfer is also possible (Battilani et al. 2006; Ikeda et al. 2000). The dog trade and transport between countries play important roles in expanding the geographical distribution of CPV-2 variants, facilitated by free movement of people and their companion animals between the European Union and other countries worldwide. A similar situation occurs in other continents, such as Asia and South America. As a result, closely related CPV-2 viral sequences have been detected across continents (Tucciarone et al. 2018). Mira et al. (2017), for example, identified CPV-2c with an Asian-origin variant in a dog transported from Thailand to Italy. Their genome analysis of amino acid changes showed that CPV-2c is more closely related to Asian than European variants. Awad et al. (2018) reported that Egyptian FPV isolates were closely related to Portuguese isolates. As a result, new variants can arise in fields where they have not been previously reported via the international transportation of animals.

Vaccines play a pivotal role in protecting dog populations against CPV infection. Updating currently available vaccines has become important because of the emergence of new variants of CPV. Some studies (Decaro & Buonavoglia 2012) have reported that CPV-2-based vaccines protect against all CPV-2 variants, while others have shown that CPV-2c was detected in dogs regularly vaccinated with vaccines containing the original CPV-2 (Ntafis et al. 2010; Pratelli et al. 2001). Geng et al. (2015) reported that dogs became infected with CPV-2 despite having been vaccinated for that virus. Thus, current vaccines may not protect sufficiently against different CPV variants. Calderon et al. (2009) showed that vaccinated dogs have become infected with CPV-2c in Argentina. More recently, Sutton et al. (2013) reported that vaccinated dogs were confirmed with severe haemorrhagic gastroenteritis caused by CPV-2c. Although the pathological and epidemiological conditions are not completely known, CPV-2c causes a more severe disease in adult dogs than CVP-2a/2b variants (Chiang et al. 2016). In Australia, CPV-2c has been detected in vaccinated dogs even though the vaccination schedule was followed completely using commercial vaccines against CPV-2b. These dogs presented with nonspecific clinical symptoms (without vomiting or with mild diarrhoea) (Woolford et al. 2017; Wilson et al. 2014). There may be a variety of reasons for vaccine failure, including improper administration techniques, inappropriate vaccine handling, maternal antibodies, the virus variant used and the degree of attenuation of the vaccine virus (Hernandez–Blanco & Catala–Lopez 2015). Monitoring both old and new CPV variants is important to understand virus evolution and develop preventive measures such as vaccines. The vaccines used and the immune status of puppies determine the vaccination efficacy (Chiang et al. 2016; Woolford et al. 2017).

Phylogenetic analysis based on partial VP2-region variants in Turkey demonstrated that, in this study, the sequences were located among viruses from other countries. The vaccine and field viruses formed distinct genetic branches. Specifically, amino acid substitutions are pivotal to genetic complexity and may result in vaccine failure and other disadvantages. New vaccines that include currently circulating strains should therefore be used to ensure appropriate and effective immunisation in Turkey.

Consequently, this study provided the first molecular identification of CPV-2c and demonstrated the circulation of CPV-2a/2b variants in the dog population of southeast Anatolia, Turkey. Moreover, this study offered the first use of RFLP and PCR/sequence analysis for the detection and characterisation of CPV-2 variants from clinical samples obtained from dogs with gastroenteritis in Turkey. Although CPV-2 is a DNA virus, the detected amino acid substitutions indicated that CPV-2 has evolved continuously. These mutations on the CPV-2 VP2 protein may cause currently used vaccines to fail. Regular epidemiological surveys and molecular studies can identify new CPV-2 genetic variants and changes. Future epidemiological and molecular surveys will help to better trace the distribution of CPV-2c and other variants in dogs in Turkey. Moreover, the use of appropriate test systems, such as serological and/or molecular tests, will reveal the real incidence of CPV-2c in field samples in Turkey. Finally, vaccination programmes and the vaccines used in dogs should be revised considering the CPV-2 variants in the field. All the obtained data will enable more effective control of CPV-2 infections in the country.
